# Improving hospital hygiene to reduce the impact of multidrug-resistant organisms in health care–a prospective controlled multicenter study

**DOI:** 10.1186/s12879-015-1184-5

**Published:** 2015-10-22

**Authors:** Miriam G. Gerlich, Jens Piegsa, Christian Schäfer, Nils-Olaf Hübner, Florian Wilke, Susanne Reuter, Georg Engel, Ralf Ewert, Franziska Claus, Claudia Hübner, Walter Ried, Steffen Flessa, Axel Kramer, Wolfgang Hoffmann

**Affiliations:** Institut für Community Medicine, Ernst-Moritz-Arndt-Universität, Ellernholzstraße 1-2, 17487 Greifswald, Germany; Institut für Hygiene und Umweltmedizin, Ernst-Moritz-Arndt-Universität, Walter-Rathenau-Straße 49a, 17475 Greifswald, Germany; Universitätsapotheke, Ernst-Moritz-Arndt-Universität, Friedrich-Ludwig-Jahn-Straße 20, 17475 Greifswald, Germany; Zentrum für Innere Medizin, Klinik und Poliklinik für Innere Medizin B, Ernst-Moritz-Arndt-Universität, Ferdinand-Sauerbruch-Straße, 17475 Greifswald, Germany; Lehrstuhl für Allgemeine Volkswirtschaftslehre und Finanzwissenschaft, Ernst-Moritz-Arndt-Universität, Friedrich-Loeffler-Straße 70, 17487 Greifswald, Germany; Lehrstuhl für Allgemeine Betriebswirtschaftslehre und Gesundheitsmanagement, Ernst-Moritz-Arndt-Universität, Friedrich-Loeffler-Straße 70, 17487 Greifswald, Germany

**Keywords:** Nosocomial infections, Multidrug-resistant organisms, MRSA, VRE, MRGN, Antibiotic use, Hospital hygiene, Costs, Health-related quality of life

## Abstract

**Background:**

Nosocomial infections are the most common complication during inpatient hospital care. An increasing proportion of these infections are caused by multidrug-resistant organisms (MDROs). This report describes an intervention study which was designed to address the practical problems encountered in trying to avoid and treat infections caused by MDROs. The aim of the HARMONIC (Harmonized Approach to avert Multidrug-resistant Organisms and Nosocomial Infections) study is to provide comprehensive support to hospitals in a defined study area in north-east Germany, to meet statutory requirements. To this end, a multimodal system of hygiene management was implemented in the participating hospitals.

**Methods/design:**

HARMONIC is a controlled intervention study conducted in eight acute care hospitals in the ‘Health Region Baltic Sea Coast’ in Germany. The intervention measures include the provision of written recommendations on methicillin-resistant Staphylococcus aureus (MRSA), vancomycin-resistant Enterococci (VRE) and multi-resistant Gram-negative bacteria (MRGN), supplemented by regional recommendations for antibiotic prescriptions. In addition, there is theoretical and practical training of health care workers (HCWs) in the prevention and handling of MDROs, as well as targeted and critically gauged applications of antibiotics.

The main outcomes of the implementation and analysis of the HARMONIC study are: (i) screening rates for MRSA, VRE and MRGN in high-risk patients, (ii) the frequency of MRSA decolonization, (iii) the level of knowledge of HCWs concerning MDROs, and (iv) specific types and amounts of antibiotics used.

The data are predominantly obtained by paper-based questionnaires and documentation sheets. A computer-assisted workflow-based documentation system was developed in order to provide support to the participating facilities. The investigation includes three nested studies on risk profiles of MDROs, health-related quality of life, and cost analysis. A six-month follow-up study investigates the quality of life after discharge, the long-term costs of the treatment of infections caused by MDROs, and the sustainability of MRSA eradication.

**Discussion:**

The aim of this study is to implement and evaluate an area-wide harmonized hygiene program to control the nosocomial spreading of MDROs. Comparability between the intervention and control group is ensured by matching the hospitals according to size (number of discharges per year / number of beds) and level of care (standard or maximum). The results of the study may provide important indications for the implementation of regional MDRO management programs.

## Background

Nosocomial infections are the most common complication that can occur during inpatient hospital care [1], and antimicrobial resistance is a serious threat to public health [2]. The Second European Point Prevalence Survey 2011–2012, involving 30 countries, showed that 6.0 % of the 231,459 surveyed patients had a healthcare-associated infection. The prevalence varied widely depending on country and type of ward, however [3]. In a sample of 9,626 patients from 46 hospitals in Germany, 5.1 % suffered from a nosocomial infection. 66 % of those infections (3.4 % of the whole sample) had arisen during the current hospital stay [4]. A significant proportion of these infections are caused by multidrug-resistant organisms (MDROs), with methicillin-resistant *Staphylococcus aureus* (MRSA) as the main cause [5, 6].

In Germany, the prevalence of MRSA has been nearly stable in recent years, with an 18 to 20 % resistance rate in clinical *Staphylococcus aureus* isolates [7]. It has, on the other hand, slightly decreased in high risk settings such as intensive care and surgical wards, where the proportion of MRSA among nosocomial *Staphylococcus aureus* infections decreased from 33 % to 27 % in the period from 2007 to 2012 [8]. In contrast, there has been a rise in the number of multiresistant Gram-negative bacteria (MRGN), such as third-generation cephalosporin (3GC)-resistant *Escherichia coli* and 3GC-resistant *Klebsiella pneumoniae* [9–12]. The prevalence of vancomycin resistance is also increasing in Germany, but this is mainly restricted to strains of *E. faecium*. The rate of vancomycin resistance in clinical *E. faecium* isolates varies between 8 and 15 %, with local and regional variability ranging from 0 % to over 30 % [13].

International studies have shown that between 10 and 70 % of nosocomial infections could have been prevented. Important factors involved in this variation were setting, study design, baseline infection rates and type of infection [14].

When planning infection control measures, it has to be taken into consideration that patients move freely between acute care and rehabilitation facilities, as well as within the ambulant sector [15]. Therefore, hospitals should not be viewed as isolated units, but rather as connected elements of a larger modular network, requiring a regional approach [16]. In the Baltic Sea Coast region, the joint research project HICARE (Health, Innovative Care And Regional Economy) was initiated in 2011 to improve control of MDROs in north-east Germany. HICARE is supported by public funds and supplemented by industrial contributions. A basic element of the HICARE project is the implementation of a multimodal hygiene program in the acute care hospitals of the region, which was developed within the framework of the HICARE project and included all German statutory specifications for infection protection. The governmental recommendations for the prevention and control of multiresistant organisms are mandatory for medical facilities, as specified by the July 2011 revision of the German *Infection Protection Act* (Infektionsschutzgesetz) [17]. To date, nationwide mandatory recommendations have been issued for MRSA [18–20] and, more recently, for MRGN [9, 21]. In addition to these two groups of causative organisms, the HICARE program includes recommendations for dealing with VRE.

To include the patient’s point of view and experience, the current study contains a patient evaluation sheet on hospital hygiene practice, and examines as well the health-related quality of life of patients with MDROs. Only few studies have been published on these aspects. Analyses of the costs and benefits of measures to reduce multidrug-resistant organisms are also scarce in Germany [22, 23].

One problem that arises with pre- and post-studies to determine the effects of intervention measures is the fact that changes in statutory requirements over time often affect the results. Therefore, the present study is designed as a prospective, controlled intervention study. Since the goal is to implement the HICARE hygiene program in all participating hospitals, those in the control group will obtain the intervention measures immediately after completion of the study phase I (delayed-intervention group; see also Fig. 2).

The primary objectives of the intervention are:

- Early detection of MDRO carriers to prevent complications and transmissions to other patients and hospital personnel through consistent hygiene measures
- Improvement in decolonization of MRSA patients
- Improvement in regional resistance profiles by a targeted and critically assessed use of antibiotics

As this study focuses on the area-wide implementation of statutory regulations, it is strictly speaking not a clinical study, and it was therefore not registered as a clinical trial. The Ethics Committee approval numbers are: BB 64/12 (Medical Committee of the University Medicine Greifswald) and A 2013–0037 (Medical Ethics Committee of the University Medicine Rostock).

## Methods and design

### Design overview and setting

**Fig. 1:**
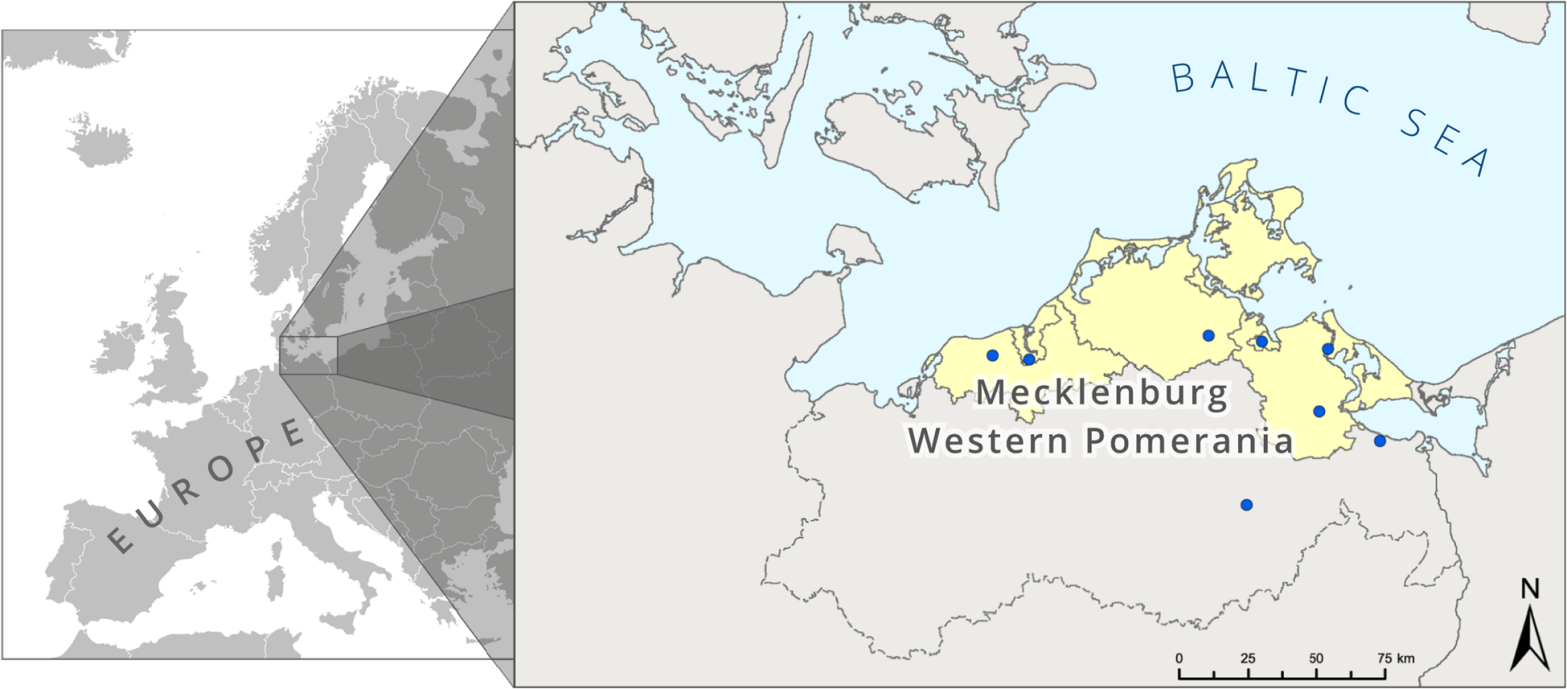
Health Region Baltic Sea Coast — hospitals participating in the study

**Fig. 2:**
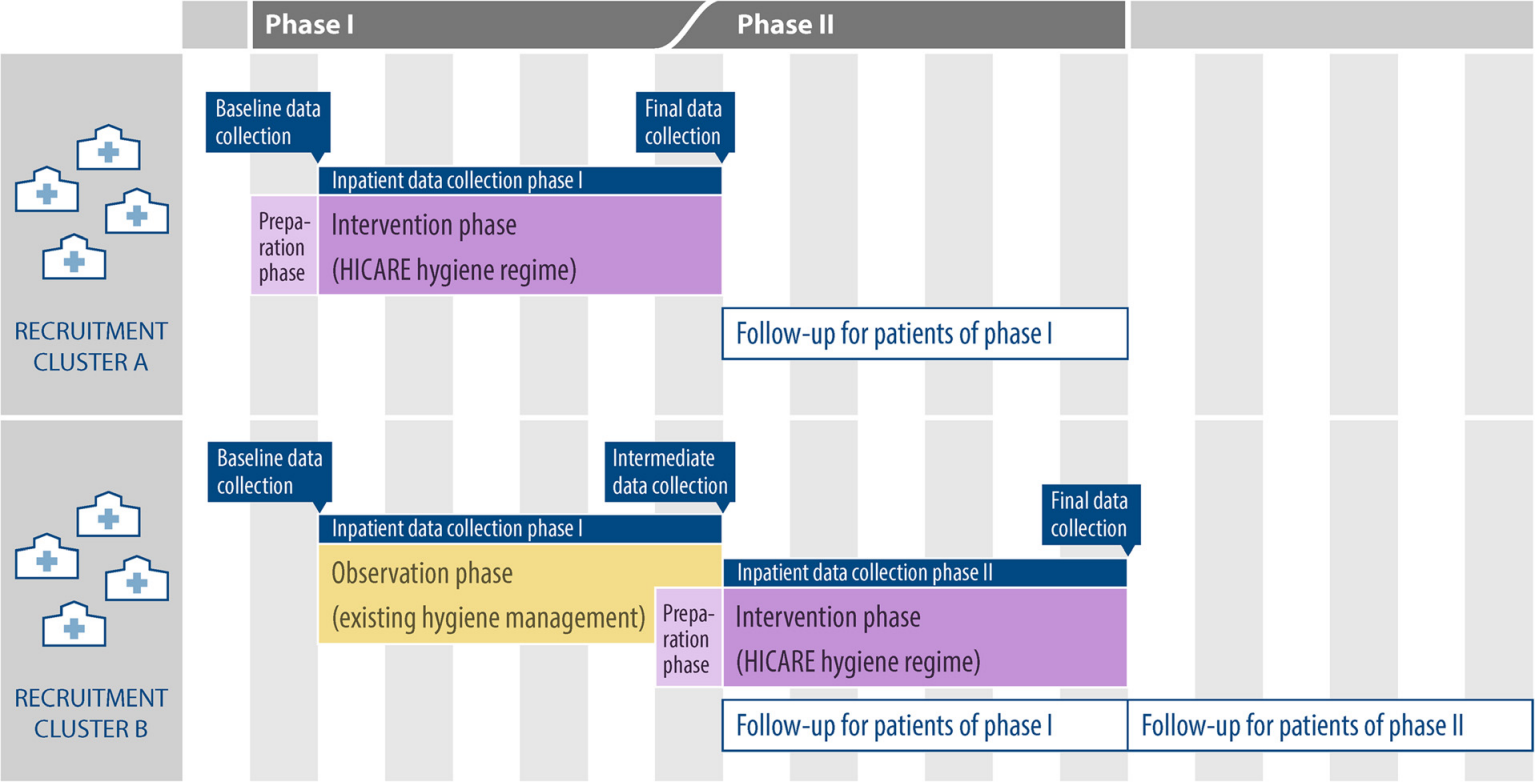
Cluster-specific study phases

A total of 12 hospitals providing acute care participate in the HICARE project. Due to lack of personnel and time resources, four of these hospitals could not participate in the HARMONIC study. Therefore, the study was conducted in eight hospitals in the Health Region Baltic Sea Coast in Germany (Fig. 1). The study was designed as an 18-month prospective intervention study, which aims at implementing a multimodal hygiene management program. The recruitment of study hospitals started right after ethics approval (29th of May 2012). Hospitals were randomized concerning the immediate implementation of the intervention program *versus* the campaign starting with a six-month delay, in four strata of two hospitals each. The strata were defined by level of care (maximum care *versus* standard care) and hospital size (>25,000; 10,000 to 25,000; 5,000 to 10,000; and < 5,000 annual discharges). Table 1 shows the characteristics used to match the participating hospitals in according pairs. The hospitals in the delayed-intervention group received the intervention measures after completion of the first phase of the study (Fig. 2). Data collection started in February 2013 and was finished for the main study in November 2014. Analysis will be finished in June 2015 and December 2015 for two of the nested studies.

**Table 1:** Characteristics used to match the study hospitals

	Number of discharges/year^a^	Number of beds^a^	Level of care^b^	Matching pair
	(Precision: 10^3^)	(Precision: 10^2^)		
Hospital 1	40,000	1,000	maximum	A
Hospital 2	35,000	900	maximum	A
Hospital 3	23,000	500	standard	B
Hospital 4	10,000	200	standard	B
Hospital 5	7,000	200	standard	C
Hospital 6	7,000	100	standard	C
Hospital 7	4,000	100	standard	D
Hospital 8	4,000	100	standard	D

^**a**^Data refer to the year 2010

^**b**^Levels of care (maximum care or standard care)

-maximum care hospitals: tertiary care; major hospitals with specific sub-specialty care

-standard care hospitals: secondary care; the range of services is restricted to prevalent conditions and a typical range of risks

Data were collected at the beginning and the end of each study phase. These data include structural data and the level of knowledge of hospital personnel with regard to MDROs. Furthermore, patients in selected wards were monitored for a period of six months in each study phase (see also Fig. 2). To maximize comparability of the participating hospitals, data were obtained for comparable subsets of three wards, each including a conservative intensive care ward, an internal medicine ward and a surgical ward. According to the specific structure and procedures of the hospital, an emergency ward was also included where applicable.

Various groups of MDROs were investigated in this study: MRSA, VRE and MRGN. Multiresistant Gram-negative organisms were defined according to the guidelines of KRINKO (German commission for hospital hygiene and infection protection) for MRGN [21]. This classifies MRGN into three groups, based on the susceptibility to four of the most clinically important groups of antibiotics (*acylureidopenicillins*, *quinolones*, 3rd gen. *cephalosporines*, *carbapenems*): 3MRGN is resistant to three of the four groups, 4MRGN is resistant to four of the four groups, and strains resistant to less than three groups are classified as non-multiresistant. The underlying mechanisms (e.g. ESBL, CPE) are not considered in this classification. Since the ability to accumulate extended-spectrum beta-lactamase (ESBL) is still partly used to categorize organisms, this was also included in the data collection.

The facilities participating in this study are acute care hospitals in the Health Region Baltic Sea Coast and adjacent hospitals, including two hospitals with maximum care status (tertiary care; major hospitals with specific sub-specialty care), and six public and private acute care hospitals with basic and standard care (secondary care; the range of services is restricted to prevalent conditions and a typical range of risks).

### Recruitment of patients and informed consent

Written informed consent was obtained for participation in this study. The patients could accept or refuse certain aspects of participating in the study: the forwarding of personal data to an independent trust agent, the pseudonymized storage of treatment data in a research database, the possibility of being recontacted at a later date for follow-up purposes, and the authorization to contact health care-related people or institutions, such as their family doctors or health insurance. Basic, anonymous information on refusal was tracked (gender and age) and considered in the analysis.

### Hypotheses

The intervention is expected to lead to:

- An increased screening rate of persons at risk for MDROs
- An increase in MRSA decolonization where indicated
- An increase in knowledge of the healthcare personnel about the prevention and handling of MDROs, e.g. treatment of infections
- A lower rate of antibiotic prescriptions
- A higher use of hand and surface disinfectants

### Intervention / hygiene measures

The HICARE intervention is based on (A) system change (hygiene regime) and (B) behavior change methods:

 (A) Existing hygiene regimes of the participating hospitals were adapted to the HICARE hygiene program by a joint coordination process (harmonized approach). A goal plan was set up to support necessary improvements, based on a comparison of the status quo and the targets of the intervention, documented via a standardized table sheet. While planning the implementation of the intervention, findings to change management in the field of hygiene measures were considered [24]. For example, it was important to continuously interact with the responsible hygiene specialists at each study site (doctors and nursing staff). These persons also acted as coordinators on site.

(B) To accomplish behavioral change, behavior-centered theoretical frameworks were taken into consideration [25–27]. Key aspects from multiple domains were addressed during conception of the intervention, such as: knowledge, skills, self-efficacy, beliefs about consequences, motivation and goals, memory, attention and decision processes, and the environmental context and resources (e.g. physical resources, management aspects). Several behavior change methods were taken into account [26, 28] and applied to the different modules of the intervention.

Evidence-based, pathogen-specific recommendations for the prevention and handling of MRSA, VRE and MRGN were drafted within the framework of the HICARE program, including governmental recommendations. The HICARE recommendations, which also include regional recommendations for the initial prescription of antibiotics for adults, represent the basis for the intervention.

The constituent parts of the intervention and the measures to promote the implementation are described in Table 2.

**Table 2:** Constituent parts of the intervention and measures to promote the implementation

Constituent parts of the intervention	Measures to promote the implementation
1. Theoretical and practical training courses for HCWs by the study team in: risk-based MDRO screening, isolation in suspected and confirmed cases of MDRO, treatment of MDRO cases including evidence-based decolonization protocol for MRSA, general measures to prevent MDROs (standard hygienic measures such as disinfection of hands and bed site surfaces, protective clothing)	For each hospital, together with the coordinator on-site, several alternative appointments were set up for each training course. Thus, it was possible that nearly all HCWs of the participating wards could join the courses. The courses were registered by the medical associations as advanced training, which adds to the attractiveness of the course.
2. Instructions on treatment of MDRO including an evidence-based decolonization protocol for MRSA ( The first line therapy for MRSA decolonization in the HARMONIC intervention was Mupirocin nasal ointment. In case of Mupirocin-resistance, polihexanide nasal ointment was used. For antisepsis of buccal cavity octenidine is used, alternatively polihexanide.)	The computer-assisted system ensures the implementation of the intervention by a step by step guidance. Pocket cards for the decolonization protocol for MRSA were provided.
3. Instruction courses by the study team for physicians on the recommendations for use of antibiotics	Several alternative appointments were offered to achieve a high participation rate. The courses were registered by the medical associations as advanced training, which adds to the attractiveness of the course.
4. Provision of detailed information material on the intervention	A folder with the course material was available on each ward. Information was additionally provided *via* a study website (separate log in for intervention hospitals).
5. Posters to intervention measures and application of study instruments	They function as a reminder.
6. Pocket cards for screening regime and decolonization protocol, see also point 2.	They function as a reminder.
7. Periodic on-site visits by the study team to provide advisory support on the implementation of the intervention measures and documentation of the improvement	The documentation was done *via* table structure and text fields. Feedback on performance was given (sources: on-site visits and monitoring process)
8. Provision of a “hotline” for questions to the intervention measures, manned by experts in hospital hygiene, who belonged to the study team	Requests of HCWs of the participating wards to single intervention measures could be answered timely. Furthermore, questions were documented anonymously and are used in the evaluation process of the study.
9. Provision of information material for patients (booklets on different MDROs)	The material supports patient information and education.

### Screening and diagnostic procedure

The following sites were screened for MRSA, according to the HARMONIC intervention: pooled nasal swab (both nostrils), throat and wounds or catheter positions if infected by bacterial culture. The laboratory diagnosis of MRSA differs in the participating hospitals, depending on availability of methods:

- PCR, if PCR is positive: confirmation by culture,
- only by culture *or*
- mainly by culture, unless a fast result by PCR is necessary.

Sites of screening for VRE include stool samples, and in exceptional cases rectal swabs. Detection of VRE is done by culture.

Sites of screening for MRGN are:

- Enterobacteria: stool sample and rectal swab; anatomic sites with former positive MRGN results; materials from potentially infected body fluids such as urine, wound secretions, tracheal secrete, blood culture and further stool samples or rectal swabs,
- Multiresistant non-fermenting organisms: throat swab, large-scale skin swab (*A. baumannii*).

Diagnostic results are obtained by culture.

### Collaboration with industry

The research institutions that organized this joint project cooperate with the healthcare management companies that take part in the HICARE project, as requested by the federal funding organization. The following materials used during the intervention phase were provided by the companies: automatic hand disinfectant dispensers, sterile water filters for intensive care wards, confectioned MRSA treatment kits for standardized decolonization, and care gowns that are impermeable for pathogens for barrier nursing.

### Outcome measures

Primary outcome:

- Screening rates for MRSA, VRE and MRGN in risk patients

Secondary outcomes:

- Frequency of MRSA decolonization upon indication
- Level of knowledge about MDROs by the nursing staff and physicians
- Types and amounts of antibiotics use
- Use of hand and surface disinfectants

### Nested studies

#### Analysis of risk profiles for multidrug-resistant organisms in patients screened for MDROs

The risk profiles of patients who tested positive for MDROs at admission was compared to patients who tested negative. Analyses will be performed for MDROs in general, as well as for individual groups of pathogens, as examined in the HARMONIC study.

#### Analysis of health-related quality of life of MDRO carriers

To define the impact of MDRO infection or carriership on quality of life, a matched analysis was performed (ratio 1:1). The health-related quality of life for patients who tested positive for MDROs will be compared to patients who tested negative and who were admitted in the participating wards of the same hospital. Matching criteria will include sex, age (+/− 5 years) and severity of illness. The patient-reported SF-36 Health Survey as modified for use in a hospital setting [29] is to be filled out shortly before discharge from the participating ward.

#### Analysis of MDRO-related hospital costs

A further study will assess the costs of MDRO management in hospitalized patients. The analysis is based on the data from the documentation sheet on the progression of MDRO status, and from the documentation sheet for nursing efforts. Additionally, lengths of hospital stay and other parameters relating to the German Diagnosis Related Group payment system will be evaluated with a focus on remuneration effects. MDRO-positive patients will be compared with MDRO-negative patients (separately for each pathogen) via matching analysis (ratio 1:3). Matching variables are the main discharge diagnosis (ICD-10) and the type of discharging ward.

### Follow-up examinations

A six-month follow-up program is planned for patients taking part in the study and who have been screened for MDRO during their hospital stay. This program is divided into three parts:

1. Health-related quality of life: quality of life survey six months after discharge from hospital, in comparison to patients who were tested negative for MDRO during their time as in-patient (control group). To this end, the modified SF-36 form will be sent by mail to the home addresses by the independent trust agency including a return mail envelope addressed to the trust agency.
2. Sustainability of MRSA decolonization: MRSA status will be determined by culture diagnosis six months after discharge from hospital. For this test, a trained nurse will visit the patient at home and take a nasal and, if requested, throat and/or wound swab. The sample will be sent to a central laboratory.
3. Health care utilization over a period of six months after discharge for patients who were screened for MDRO during their hospital stay. The data for positive patients are compared to those with negative results. For this reason, a patient-reported questionnaire including a reply-paid envelope will be sent to the home address by the independent trust agency. Secondary data of health insurance companies will be analyzed as well.

In each case, the accompanying informed consent policies have to be agreed by the patient.

### Methods of data collection and variables

1. Baseline data collection conducted with participating hospitals included a questionnaire to assess structural parameters (e.g. number of beds/cases/single rooms, staffing ratio, staff level of qualification, prevalence of advanced training in hygiene), and process parameters (procedures of registering, documentation and reporting of MDROs). At the end of each study phase (Fig. 2), potential changes in structural or process parameters were documented, together with use of antibiotics and disinfectants for the given period.
2. Data on the use of antibiotics and disinfectants over the three years prior to this study, as well as test utilization and resulting positive MDRO rates, were documented by the study team.
3. The level of knowledge of the nursing staff and physicians on MDROs was assessed by a multiple choice test at the beginning of the instructional courses.
4. Structured questionnaires and documentation forms were used during the intervention period, and, for the delayed-intervention group only, during the observation period, identical for all hospitals (Table 3).

**Table 3:** Documents used for data acquisition during the intervention and observation phase

Documents used in the intervention and observation phase	Description of data collected	Document to be filled out by
Patient admission sheet	Risk factors for MDRO colonization or infection	Health care workers during anamnesis, or, alternatively, by the patient (with help of staff)
Documentation sheet on the progression of MDRO status including transition section^**a**^ for the physician responsible for subsequent treatment	Admission and discharge data, sample taking and diagnosis, where applicable isolation measures, control swab test, time of infection, information for physician responsible for subsequent treatment	Health care workers
Documentation sheet for nursing efforts for MDRO^b^ infections/colonizations	Nursing workload in minutes	Nursing staff
Patient evaluation sheet	Subjective evaluation of hygiene measures in the hospital	Patient
Quality of life questionnaire (modified version of the SF-36)	Health-related quality of life in the last seven days	Patient

^**a**^This form differs slightly for the delayed intervention group and the immediate intervention group. In order to exert as little influence as possible on the documentation of previous hygiene management programs, the form for the delayed intervention group does not include the transition section for the physician responsible for subsequent treatment

^b^also relevant for reimbursement by the statutory health insurance

Note: Documents can be downloaded from: http://www2.medizin.uni-greifswald.de/icm/index.php?id=hicare

**Fig. 3:**
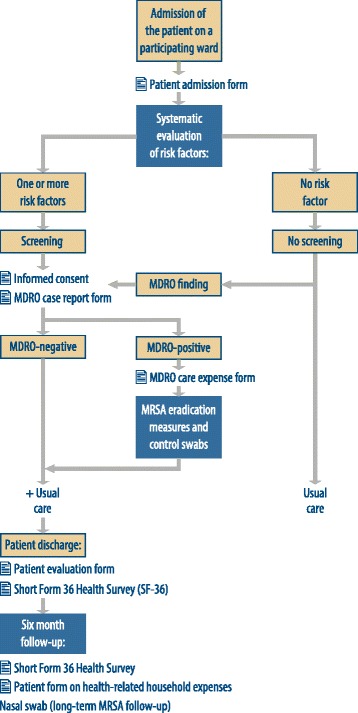
Patient-specific study workflow — intervention group

The usage of the questionnaires for the collection of patient data is shown in the flow chart in Fig. 3. Participation was only requested if the patient was expected to remain in the ward for at least 48 hours. The patient admission sheet and both MDRO documentation sheets were designed to replace the previous documentation used by the hospitals. This measure also avoids time and effort of double documentation. The treatment sheets are designed such that the original has space for a label with the patient’s identification and is included in the patient’s file as treatment documentation after the patient is discharged. For the sake of data privacy protection, the carbon copy that is used for our concomitant research will not have a label with patient identifying data. Both the original and the carbon copy are provided with a document barcode, allowing a mapping of multiple documents to a single pseudonym and preserving anonymity for non-consenters.

A workflow-oriented documentation system was developed that is made available to the participating wards on a tablet PC, a *Medical Clinical Assistant* (MCA) with an integrated barcode scanner and a touch screen that can be disinfected. This system serves two purposes:

i. Support during the study
  Upon admission of a patient who is expected to spend at least 48 hours on the ward, the basic data (given name, surname, and date of birth) are entered into the system. The system then guides the staff through the sequence required by the study according to the individual patient, indicating which forms are to be filled out at which time. After completion, the forms respectively their copies are dropped into a special mailbox and are subsequently transferred to a central data management. Patient permissions are collected in a separate container and are transferred to the independent trust agent. Staff members from the participating wards are trained in how to use the documentation system by HARMONIC study personnel.
ii. Data privacy protection
  Patient data entered into the workflow-oriented documentation system are encrypted by hospital-specific private keys and only visible to the responsible HCWs. To allow the individual forms and copies to be correlated to a specific person, each sheet of the form contains an ID that can be read out by the barcode scanner integrated in the MCA. This system provides the relational mapping of the individual sheet IDs to the participant ID, while assuring the data privacy protection.

### Data management and protection procedures

#### Monitoring procedures

The workflow-oriented documentation system was developed as a web-based solution. This allows real-time monitoring of the individual paper forms, so that any problem arising with the use of the forms can be quickly detected and solved. The monitoring dashboard contains aggregated data on the usage of the forms, but does not show any data about individuals.

#### Data protection procedures

**Fig. 4:**
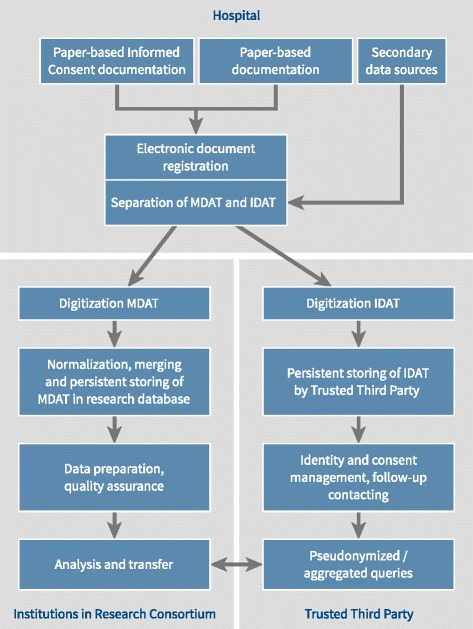
Data management activities and responsibilities

A data protection plan was developed for the HARMONIC study in conjunction with the State Commissioner for Data Protection and Freedom of Information. If a patient gives permission to store his or her treatment data under a pseudonym, the personal identifying data (name, address) is collected on the informed consent document. Management of the consent documents is performed by an independent trust agent. Pseudonymized medical data are stored in a research database separated from the identifying data in an approach (Fig. 4) that follows the recommendations of the “Generic solutions for data protection in medical research networks – model B”, developed by the umbrella organization for networked medical research in Germany (TMF) [30, 31]. These recommendations were accepted by the German conference of federal and state data protection commissioners.

The study data originate from primary and secondary data sources and are registered using document IDs, serving as study pseudonyms. Identifying data and medical data are processed as shown in Fig. 4. In the research database, only double pseudonymized data are stored, which prevents re-identification of the patient while analyzing the data. If necessary, further measures will be taken to impede re-identification, e.g. aggregation or precision reduction of informative variables such as age.

If no informed consent can be obtained, no identifying data will be transmitted. In these cases, a follow-up is not possible.

### Data analysis

#### Sample size

According to German screening recommendations on MRSA, about 40 % of patients admitted in acute hospitals in Germany have risk factors for MRSA [32]. It has been shown that screening recommendations are not followed completely, despite their obligatory character [33]. Based on information solicited with our institutional questionnaire, not all of the participating hospitals had fully adapted to the recommendations at baseline. Thus, as basis for sample size calculations, our intervention was assumed to improve the screening rate in MRSA risk patients from 60 % (control group) to 90 % (intervention group). The expected sample of > 1000 patients in each group results in a very high statistical power (approaching 1.0) for this study (type-I risk: 0.05).

With an observation period of six months for each of the 24 participating wards, at least 5,000 patient admissions with an anticipated stay over 48 hours are expected. Based on previous examinations [7, 8, 34, 35], an MRSA prevalence at admission of approximately 1 to 3 % is anticipated. Accordingly, 50 to 150 MRSA positive patients would be eligible for follow-up examinations.

#### Data analysis

To evaluate the effect of the outcome measures, a hierarchical logistic regression model for the log-odds of a patient’s chance to be screened for either MRSA, VRE, or MRGN was formulated as follows:

(level 1):

$$ \mathrm{logit}{\left( antibiogram=1\right)}_{ij}={a}_j+{\beta}_1{X}_{1ij}+{\beta}_2{X}_{2ij}+\cdots +{\varepsilon}_{ij} $$

with

$$ \begin{array}{l}\mathrm{i}:=1\kern0.28em \mathrm{t}\mathrm{o}\kern0.28em \mathrm{k}\kern4.5em \mathrm{patient}\kern.3em \mathrm{i}\mathrm{ndex}\kern1em \\ {}\mathrm{j}:=1\kern0.28em \mathrm{t}\mathrm{o}\kern0.24em 8\kern4.5em \mathrm{ID}\kern.3em \mathrm{number}\kern0.28em \mathrm{o}\mathrm{f}\kern0.28em \mathrm{hospital}\kern1em \\ {}{\mathrm{X}}_1\kern0.28em \mathrm{t}\mathrm{o}\kern0.28em {\mathrm{X}}_{\mathrm{m}}\kern4.8em \mathrm{i}\mathrm{ndividual}\kern.3em \mathrm{patient}\kern0.28em \mathrm{c}\mathrm{hartacteristics},\mathrm{such}\kern.2em \mathrm{a}\mathrm{s}\kern.2em \mathrm{a}\mathrm{ge},\mathrm{sex},\kern.2em \mathrm{s}\mathrm{urgical}\kern0.28em \mathrm{t}\mathrm{reatment}\kern.2em \mathrm{e}\mathrm{t}\mathrm{c}.\kern1em \end{array} $$

As the intervention program was implemented on the level of participating hospitals, the following level-2 equation (random intercept model) was a priori specified for statistical analysis:

$$ {a}_j={\gamma}_{00}+{\gamma}_{01}{\zeta}_{1j}+{\gamma}_{02}{\zeta}_{2j}+\cdots +{\omega}_j $$

with γ_00_ representing the global base rate parameter for screening tests, and γ_0j_ hospital-specific additions depending on the intervention group (ζ_1_ = 1 for intervention period, 0 otherwise), hospital capacity (ζ_2j_ measured on metric scale level) or similar. To test the main hypothesis, the significance of parameter γ_01_ (deviation from zero) will be tested. Control for potential center effects is exerted by introduction of the ω_j_ center-specific error terms of the intercept parameter.

It will be decided during the model selection process whether or not other variables on the hospital level are to be introduced in separate level-2 equations as so-called random slope-effects (e.g. hospital-specific tendencies towards testing older patients more frequently).

For an assessment of the differences between the matched groups in the costs of MDRO management and treatment, as well as in health-related quality of life, *t*-tests will be applied. In order to control for the influence of covariates, likewise multivariate analysis methods and, in particular, logistic regression analysis will be applied to address selected issues.

### Ethical approval

The study protocol including the nested studies was endorsed by the Medical Ethics Committee of the University Medicine Greifswald (BB 64/12) and by the Medical Ethics Committee of the University Medicine Rostock (A 2013–0037).

## Discussion

The development and implementation of infection control programs is becoming increasingly important, since medical care has become more complex and antimicrobial resistance has increased [15]. Different management approaches play an important role in preventing nosocomial infections with MDROs, including screening of high-risk patients, barrier precautions, compulsory hand hygiene, cleaning and disinfection, and surveillance of infections [15, 22]. Furthermore, there is an increased interest in MRSA decolonization measures [15, 36]. Study results show that nasal decolonization with Mupirocin, in combination with other substances, may lead to a reduction of MRSA infections. However, long-term effects have so far barely been investigated [22]. Therefore, the assessment of the sustainability of decolonization measures is one important aspect in the follow-up measures of our investigation.

A main goal of the HICARE joint research project is to integrate fragmented compulsory hygiene measures into a comprehensive hygiene concept. This concept, in addition to expanding upon basic hygiene, encompasses specific measures for the prevention and treatment of MRSA, VRE and MRGN. The project will be implemented and evaluated for the model region within the framework of the HARMONIC study. Since not all facets can be considered in the evaluation due to personnel and financial constraints, the screening rate of high risk patients and frequency of MRSA decolonization treatment upon indication are defined as main study outcomes. Hence, early recognition of MDROs and professional treatment are the main objectives of the HICARE project.

While implementing the prevention strategies, specific conditions of the participating hospitals are considered. In addition to optimizing processes and providing advanced training for HCWs, a further goal is to provide patients with information on how to prevent infections (e.g. correct disinfection of hands during their stay in the hospital).

### Strengths and limitations

An intervention with such a wide approach has strengths, but it also has limitations. One strength of this project is that by contributing to the regional management of MDROs, other regions can profit from the findings on the implementation of the structure and processes. On the other hand, possible effects of the study cannot be ascribed to individual interventions, but rather are the result of the total intervention program combined with changes motivated by external factors, including campaigns initiated by the local government, or regional public health offices. The nested studies are designed to provide insight into the cost-benefit aspects, the health-related quality of life, as well as the risk profiles of the various MDROs. The HARMONIC study has a highly interdisciplinary basis, providing insight into medical and nursing care, epidemiology and health economics. These may provide a basis for decisions in health care policies.

A limitation of our study is that it was not possible to provide direct financial support to the hospitals for the additional cost incurred by the implementation of the program. It was therefore necessary to find a workable model that could be implemented within the framework of daily hospital routine. Consequently, it was necessary for certain aspects to fall back on the current documentation of the hospital; e.g. for microbiologic results, it was necessary to rely on the hospital laboratory rather than on the installation of a central diagnostics. This could, however, prove to be a strength in the long run, as we had to find solutions that are likely to be sustainable under routine conditions.

The following factors were taken into consideration in the conception of the HARMONIC study:

- Limitations of staffing resources (e.g. for documentation of hygiene measures)
- Limitations of financial resources (e.g. for additional screening examinations)
- Structural factors of the hospitals (e.g. number of single rooms, staff / patient ratio)

The HICARE hygiene management system was tailored to the possibilities and requirements of the individual hospitals by a mutual coordination process. The results of this coordination are documented in detail and will be taken into consideration in the evaluation of the project. This approach was designed to achieve a regional standard for hygiene recommendations based on scientific evidence that is affordable for the hospitals. Close collaboration with the staff responsible for hygiene and with the policy makers of the hospitals should ensure that these hygiene measures will be sustainably implemented after the end of the study. Despite the limitations, we believe that with the design of this study and the chosen parameters, the groups will be comparable.

An important topic would be the incidence of MDROs before and after the intervention. Unfortunately, our investigation does not collect sufficient data to analyze questions referring to MDRO incidence. Due to limited financial resources, as mentioned above, we were not able to conduct additional diagnostics to analyze this outcome.

In 2011, parts of the German infection protection act were changed and further complemented in 2013. The law says that the heads of defined medical institutions have to ensure that measures according to the current standard of medical science have to be applied to prevent the spread of nosocomial infections, especially those with multidrug resistance. It is assumed that this standard is applied when the recommendations of the “commission for hospital hygiene and infection protection” are considered. In the HARMONIC study, we support regional acute hospitals to implement those recommendations timely and effectively, implementing consistent processes that can be adapted to local challenges, and managed and monitored. Nevertheless, at least some of the control hospitals are assumed to have already implemented the national recommendations. However, the extent may differ due to limited strategic resources, so an improvement as a result of our intervention is expected.

### Implications

The results of the HARMONIC study are expected to have a direct impact on clinical practice, improving the effectiveness of targeted screening and multibarrier measurements. Further goals are:

- Improvements and standardization of the MDRO patient transfer process to the physician responsible for further treatment (usually the patient’s general practitioner)
- Improvement of the practice of antibiotics prescription
- Improvement of the quality of care (e.g. treatment of MRSA)
- Informing patients about MDROs

Hospitals are presently facing increasing competition. Participation in programs such as these allow a facility to demonstrate that they are actively taking action against the proliferation of MDROs, thereby providing protection both to patients and health care workers.

The results of this study will indicate whether or not it is worth the effort, both economically and from the standpoint of patient safety and satisfaction, to continue with this hygiene regimen after the end of the HARMONIC study.

The website www.hicare.de provides information on the HARMONIC study and provides links to additional information on the HICARE joint research project.

## Abbreviations

ESBL: Extended-spectrum beta-lactamase producing bacteria
HARMONIC: Harmonized Approach to Avert Multiresistant Organisms and Nosocomial Infections
HCWs: Health Care Workers
MCA: Medical Clinical Assistant
MDROs: Multidrug-resistant organisms
MRSA: Methicillin-resistant Staphylococcus aureus
VRE: Vancomycin-resistant Enterococcus
MRGN: Multi-resistant Gram-negative bacteria (also MDRGN: multidrug-resistant Gram-negative bacteria)
SF-36: The Short Form 36 Health Survey
WHO: World Health Organization
